# Generalizing Terwilliger's likelihood approach: a new score statistic to test for genetic association

**DOI:** 10.1186/1471-2156-8-63

**Published:** 2007-09-24

**Authors:** Rachid el Galta, Shirley Uitte de Willige, Marieke CH de Visser, Quinta Helmer, Li Hsu, Jeanine J Houwing-Duistermaat

**Affiliations:** 1 Department of Medical Statistics and Bioinformatics, Leiden University Medical Center, PO Box 9600, 2300 RC Leiden, The Netherlands; 2 Department of Thrombosis and Hemostasis, Leiden University Medical Center, Leiden, The Netherlands; 3 Division of Public Health Sciences, Fred Hutchinson Cancer Research Center, Seattle, Washington, USA

## Abstract

**Background:**

In this paper, we propose a one degree of freedom test for association between a candidate gene and a binary trait. This method is a generalization of Terwilliger's likelihood ratio statistic and is especially powerful for the situation of one associated haplotype. As an alternative to the likelihood ratio statistic, we derive a score statistic, which has a tractable expression. For haplotype analysis, we assume that phase is known.

**Results:**

By means of a simulation study, we compare the performance of the score statistic to Pearson's chi-square statistic and the likelihood ratio statistic proposed by Terwilliger. We illustrate the method on three candidate genes studied in the Leiden Thrombophilia Study.

**Conclusion:**

We conclude that the statistic follows a chi square distribution under the null hypothesis and that the score statistic is more powerful than Terwilliger's likelihood ratio statistic when the associated haplotype has frequency between 0.1 and 0.4 and has a small impact on the studied disorder. With regard to Pearson's chi-square statistic, the score statistic has more power when the associated haplotype has frequency above 0.2 and the number of variants is above five.

## Background

Haplotype analysis is a popular strategy to analyze multiple single nucleotide polymorphisms (SNPs) typed within one candidate gene [1,2]. Most of the genetic variation of a gene is described by 4 to 8 relatively common haplotypes (frequencies > 0.05). To compare the distribution of observed haplotype frequencies between cases and controls, the global test statistic of Schaid et al. [3] can be used when haplotype ambiguity exists. When no uncertainty in phase exists, for example in regions with little recombinations, the simple Pearson's chi square statistic is used to test for association. Both statistics test for any difference in haplotype frequencies between cases and controls and the degrees of freedom depend on the number of observed haplotypes. However, researchers often aim to identify one ancestral haplotype [4]. For this situation one may consider a test which summarizes the difference between the null hypothesis and the alternative hypothesis in one parameter, yielding a more powerful one degree of freedom test. This paper aims to derive such a method.

For multi-allelic markers, Terwilliger [5] proposed a model with one parameter (*λ*) for the excess frequency of the associated allele in the cases. To test for association he proposed a likelihood ratio test, i.e. comparing the likelihood under the alternative to the likelihood under no association, for testing. Since it is unknown which allele is associated, the likelihood is a weighted sum of conditional likelihoods of the data given that the allele is associated over the observed marker alleles. As for weights Terwilliger [5] proposed to use the allele frequencies in the population from which the cases and controls were sampled. Thus the most common allele is assigned the highest weight in the analysis. However, for haplotype analysis it is usually not the most common haplotype in the population which is associated to the disease. Therefore in this paper, we propose a model which assigns constant weight to each haplotype. We derive a score statistic from the likelihood of this model.

The log likelihood function of Terwilliger's model also appears to have some unusual statistical features. For example, the allele frequencies that are used as weights are also unknown parameters in the conditional likelihood functions. Maximizing the log likelihood function appears not always straightforward. Further its distribution under the null hypothesis is unknown. Terwilliger proposed to approximate the distribution of the likelihood ratio (*TLR*) statistic under the null hypothesis with the 50 : 50 mixture of chi square distributions of null and one degrees of freedom. This distribution appeared to yield conservative p-values [6]. Another property of the likelihood function is that the score function, the first derivative of the log likelihood function with respect to *λ *evaluated at *λ *= 0 is a constant zero for any observed data for the weights that Terwilliger proposed. Hence the corresponding score statistic does not exist. Score statistics are often preferred over likelihood ratio statistics, because they have often a tractable expression which provides insight in the data and are robust against model deviations [7]. When constant weights instead of allele frequencies as weights are used the score statistic exists. Under the null hypothesis, the statistic follows a normal distribution.

In this paper we derive a method for the extreme situation of phase unambiguity, which means that we study genetic regions with almost no recombination [8]. This assumption is reasonable for many genes, for example fibrinogen alpha, beta and gamma [9] and CRP [10]. When some uncertainty about phase exists, the p-values should be adapted. Our program [11] is able to compute valid p-values for the situation of phase ambiguity by permutation of case and control status. The program calls the publicly available software TAGSNPS [8] to derive the haplotypes in each permutation step (see also Becker et al. [12] for similar methods).

The methods derived in this paper are meant to detect a common haplotype. In general genetic association studies have power to detect common variants. Thus these methods should detect associations under the common disease-common variant hypothesis. However, it is also possible that a rare causal mutation is sometimes present on the identified haplotype. For example Hylckama Vlieg et al. [13] detected a common haplotype that was overrepresented in patients with deep venous thrombosis compared to healthy controls. Many homozygous carriers of this haplotype carried the factor V Leiden mutation, the most important genetic risk factor for deep venous thrombosis, which had been discovered before by other methods [14]. Thus if homozygous carriers of the associated haplotype were sequenced the factor V Leiden mutation would have been detected.

We illustrate the method by an association analysis of three candidate genes, fibrinogen alpha (FGA), beta (FGB) and gamma (FGA)in the Leiden Thrombophilia Study (LETS) [9]. In these data, no phase ambiguity was present and the pair of haplotypes could be derived for each individual. The number of common haplotypes varied from 3 to 5. The frequency of the associated haplotype varied from 0.2 to 0.3. In this paper, we consider the score statistic as an alternative to the classical chi-square and the original TLR statistic of [5] in the context of haplotypes. We also include the likelihood ratio statistic corresponding to the log likelihood using equal weights. We carried out a simulation study to examine the performance of the four statistics under the null hypothesis and to compare the power of the four statistics under various alternatives. Finally we illustrate the proposed statistics by analysis of the haplotype distributions of the FGG, FGB and FGA genes.

## Results

### Simulation study

By means of a simulation study we compared the performance of the new score statistic S^1m
 MathType@MTEF@5@5@+=feaafiart1ev1aaatCvAUfKttLearuWrP9MDH5MBPbIqV92AaeXatLxBI9gBaebbnrfifHhDYfgasaacH8akY=wiFfYdH8Gipec8Eeeu0xXdbba9frFj0=OqFfea0dXdd9vqai=hGuQ8kuc9pgc9s8qqaq=dirpe0xb9q8qiLsFr0=vr0=vr0dc8meaabaqaciaacaGaaeqabaqabeGadaaakeaacuWGtbWugaqcamaaBaaaleaadaWcaaqaaiabigdaXaqaaiabd2gaTbaaaeqaaaaa@307A@ with Pearson chi squared *χ*^2 ^and the Terwilliger's likelihood ratio with weights equal to *p*_*j*_'s (*TLR*). We also considered a likelihood ratio test with equal weights (*LR*). We first evaluated the type I error rates of the test statistics. For the score statistic we used the chi square distribution with one degree of freedom to approximate the distribution under the null hypothesis. For the *LR *and *TLR *statistics we used the 50:50 mixture of two chi squares with zero and one degree of freedom. We generated 10,000 samples of 200 case chromosomes and 200 control chromosomes from the multinomial distributions with probabilities *p*_1_⋯*p*_*m *_for *m *equal to 4, 5, 8, 10, 15 and 20 haplotypes. Similar to the simulation described by Terwilliger, the frequency of the most common haplotype, *p*_1_, was set to 0.5, whereas the remaining haplotypes were equally frequent (0.5/(*m *- 1)). The results are shown in the left columns of Table 1.

**Table 1:** Type I error rates and power of the statistics *X*^2^, S^1m
 MathType@MTEF@5@5@+=feaafiart1ev1aaatCvAUfKttLearuWrP9MDH5MBPbIqV92AaeXatLxBI9gBaebbnrfifHhDYfgasaacH8akY=wiFfYdH8Gipec8Eeeu0xXdbba9frFj0=OqFfea0dXdd9vqai=hGuQ8kuc9pgc9s8qqaq=dirpe0xb9q8qiLsFr0=vr0=vr0dc8meaabaqaciaacaGaaeqabaqabeGadaaakeaacuWGtbWugaqcamaaBaaaleaadaWcaaqaaiabigdaXaqaaiabd2gaTbaaaeqaaaaa@307A@, LR, TLR

		type I error rate				power when *λ *= 0.5			
*m*	nominal	*X*^2^	S^1m MathType@MTEF@5@5@+=feaafiart1ev1aaatCvAUfKttLearuWrP9MDH5MBPbIqV92AaeXatLxBI9gBaebbnrfifHhDYfgasaacH8akY=wiFfYdH8Gipec8Eeeu0xXdbba9frFj0=OqFfea0dXdd9vqai=hGuQ8kuc9pgc9s8qqaq=dirpe0xb9q8qiLsFr0=vr0=vr0dc8meaabaqaciaacaGaaeqabaqabeGadaaakeaacuWGtbWugaqcamaaBaaaleaadaWcaaqaaiabigdaXaqaaiabd2gaTbaaaeqaaaaa@307A@	LR	TLR	*X*^2^	S^1m MathType@MTEF@5@5@+=feaafiart1ev1aaatCvAUfKttLearuWrP9MDH5MBPbIqV92AaeXatLxBI9gBaebbnrfifHhDYfgasaacH8akY=wiFfYdH8Gipec8Eeeu0xXdbba9frFj0=OqFfea0dXdd9vqai=hGuQ8kuc9pgc9s8qqaq=dirpe0xb9q8qiLsFr0=vr0=vr0dc8meaabaqaciaacaGaaeqabaqabeGadaaakeaacuWGtbWugaqcamaaBaaaleaadaWcaaqaaiabigdaXaqaaiabd2gaTbaaaeqaaaaa@307A@	LR	TLR

4	0.05	0.053	0.052	0.042	0.032	0.91	0.94	0.91	0.95
4	0.01	0.009	0.010	0.010	0.007	0.78	0.84	0.80	0.86
4	0.001	0.001	0.001	0.001	0.000	0.53	0.62	0.56	0.65
5	0.05	0.053	0.048	0.040	0.032	0.88	0.94	0.89	0.95
5	0.01	0.010	0.010	0.010	0.007	0.71	0.83	0.77	0.87
5	0.001	0.001	0.001	0.001	0.000	0.44	0.59	0.53	0.64
8	0.05	0.048	0.049	0.038	0.035	0.77	0.92	0.86	0.95
8	0.01	0.008	0.010	0.008	0.004	0.53	0.80	0.74	0.86
8	0.001	0.001	0.000	0.001	0.000	0.25	0.54	0.50	0.65
10	0.05	0.045	0.051	0.034	0.023	0.70	0.90	0.84	0.95
10	0.01	0.006	0.008	0.008	0.003	0.43	0.76	0.70	0.85
10	0.001	0.000	0.001	0.000	0.000	0.18	0.49	0.47	0.60
15	0.05	0.043	0.048	0.041	0.020	0.58	0.86	0.80	0.95
15	0.01	0.007	0.010	0.010	0.003	0.31	0.69	0.65	0.85
15	0.001	0.000	0.001	0.002	0.000	0.09	0.42	0.42	0.63
20	0.05	0.043	0.052	0.045	0.021	0.48	0.84	0.77	0.95
20	0.01	0.005	0.011	0.010	0.004	0.20	0.64	0.62	0.84
20	0.001	0.000	0.001	0.002	0.000	0.04	0.35	0.39	0.60

For all *m*, the type I error rates of the score statistic S^1m
 MathType@MTEF@5@5@+=feaafiart1ev1aaatCvAUfKttLearuWrP9MDH5MBPbIqV92AaeXatLxBI9gBaebbnrfifHhDYfgasaacH8akY=wiFfYdH8Gipec8Eeeu0xXdbba9frFj0=OqFfea0dXdd9vqai=hGuQ8kuc9pgc9s8qqaq=dirpe0xb9q8qiLsFr0=vr0=vr0dc8meaabaqaciaacaGaaeqabaqabeGadaaakeaacuWGtbWugaqcamaaBaaaleaadaWcaaqaaiabigdaXaqaaiabd2gaTbaaaeqaaaaa@307A@ were maintained at the nominal error rate. For *m *< 10, the type I error rates of Pearson's chi square corresponded to the nominal level, but became conservative for larger *m *due to sparse data. For all *m *considered, the type I error rates for the *TLR *statistic were conservative (< 0.03). The type I error rates for the *LR *statistic were also somewhat small (≈ 0.04), but were better than that of the *TLR *statistic.

To study the power of the statistics, we generated 10,000 samples of 100 case chromosomes and 100 control chromosomes from the multinomial distributions with probabilities *p*_1_(1 - *λ*) + *λ *and *p*_*j*_(1 - *λ*) for *j *= 2⋯*m *for cases and *p*_*j *_*j *= 1⋯*m *for controls, respectively. First, we considered the model used by Terwilliger [5]. The most common haplotype frequency *p*_1 _in controls was again set to 0.5 and this haplotype was more frequent in cases. The parameter *λ *was fixed to 0.5 which corresponds to a haplotype frequency of 0.75 in the cases. The number of haplotypes *m *was again set to 4, 5, 8, 10, 15 and 20. The results are shown in the right columns of Table 1.

For *m *< 8, the power of Pearson chi square statistic was good. The S^1m
 MathType@MTEF@5@5@+=feaafiart1ev1aaatCvAUfKttLearuWrP9MDH5MBPbIqV92AaeXatLxBI9gBaebbnrfifHhDYfgasaacH8akY=wiFfYdH8Gipec8Eeeu0xXdbba9frFj0=OqFfea0dXdd9vqai=hGuQ8kuc9pgc9s8qqaq=dirpe0xb9q8qiLsFr0=vr0=vr0dc8meaabaqaciaacaGaaeqabaqabeGadaaakeaacuWGtbWugaqcamaaBaaaleaadaWcaaqaaiabigdaXaqaaiabd2gaTbaaaeqaaaaa@307A@ performed better than the *LR *statistic with equal weights. The power of the score statistic S^1m
 MathType@MTEF@5@5@+=feaafiart1ev1aaatCvAUfKttLearuWrP9MDH5MBPbIqV92AaeXatLxBI9gBaebbnrfifHhDYfgasaacH8akY=wiFfYdH8Gipec8Eeeu0xXdbba9frFj0=OqFfea0dXdd9vqai=hGuQ8kuc9pgc9s8qqaq=dirpe0xb9q8qiLsFr0=vr0=vr0dc8meaabaqaciaacaGaaeqabaqabeGadaaakeaacuWGtbWugaqcamaaBaaaleaadaWcaaqaaiabigdaXaqaaiabd2gaTbaaaeqaaaaa@307A@ was similar to the power of *TLR *statistic, for *m *≤ 8. For *m *> 8, the *TLR *statistic had higher power than S^1m
 MathType@MTEF@5@5@+=feaafiart1ev1aaatCvAUfKttLearuWrP9MDH5MBPbIqV92AaeXatLxBI9gBaebbnrfifHhDYfgasaacH8akY=wiFfYdH8Gipec8Eeeu0xXdbba9frFj0=OqFfea0dXdd9vqai=hGuQ8kuc9pgc9s8qqaq=dirpe0xb9q8qiLsFr0=vr0=vr0dc8meaabaqaciaacaGaaeqabaqabeGadaaakeaacuWGtbWugaqcamaaBaaaleaadaWcaaqaaiabigdaXaqaaiabd2gaTbaaaeqaaaaa@307A@. For *m *> 8, the power of the score statistic S^1m
 MathType@MTEF@5@5@+=feaafiart1ev1aaatCvAUfKttLearuWrP9MDH5MBPbIqV92AaeXatLxBI9gBaebbnrfifHhDYfgasaacH8akY=wiFfYdH8Gipec8Eeeu0xXdbba9frFj0=OqFfea0dXdd9vqai=hGuQ8kuc9pgc9s8qqaq=dirpe0xb9q8qiLsFr0=vr0=vr0dc8meaabaqaciaacaGaaeqabaqabeGadaaakeaacuWGtbWugaqcamaaBaaaleaadaWcaaqaaiabigdaXaqaaiabd2gaTbaaaeqaaaaa@307A@ is smaller, because the weight 1/*m *is small and the frequencies of the non associated haplotypes are too small, yielding a large variance of the score statistic (see formula 3). Because the *LR *statistic appeared to perform worse than both S^1m
 MathType@MTEF@5@5@+=feaafiart1ev1aaatCvAUfKttLearuWrP9MDH5MBPbIqV92AaeXatLxBI9gBaebbnrfifHhDYfgasaacH8akY=wiFfYdH8Gipec8Eeeu0xXdbba9frFj0=OqFfea0dXdd9vqai=hGuQ8kuc9pgc9s8qqaq=dirpe0xb9q8qiLsFr0=vr0=vr0dc8meaabaqaciaacaGaaeqabaqabeGadaaakeaacuWGtbWugaqcamaaBaaaleaadaWcaaqaaiabigdaXaqaaiabd2gaTbaaaeqaaaaa@307A@ and *TLR*, we did not consider this statistic in the following simulations.

**Figure 1:**
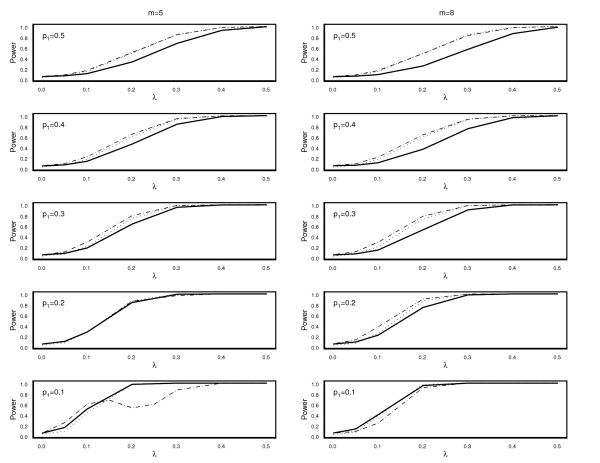
Power curves of *X*^2 ^(^____^), S^1m
 MathType@MTEF@5@5@+=feaafiart1ev1aaatCvAUfKttLearuWrP9MDH5MBPbIqV92AaeXatLxBI9gBaebbnrfifHhDYfgasaacH8akY=wiFfYdH8Gipec8Eeeu0xXdbba9frFj0=OqFfea0dXdd9vqai=hGuQ8kuc9pgc9s8qqaq=dirpe0xb9q8qiLsFr0=vr0=vr0dc8meaabaqaciaacaGaaeqabaqabeGadaaakeaacuWGtbWugaqcamaaBaaaleaadaWcaaqaaiabigdaXaqaaiabd2gaTbaaaeqaaaaa@307A@ (-·-·) and *TLR *(·····) as function of *λ *for various values of the frequency of the associated variant *p*_1 _= 0.1, 0.2, 0.3, 0.4, 0.5 and *m *= 5 (left column), and *m *= 8 (right column).

Second, we studied the power of the Pearson's chi square, S^1m
 MathType@MTEF@5@5@+=feaafiart1ev1aaatCvAUfKttLearuWrP9MDH5MBPbIqV92AaeXatLxBI9gBaebbnrfifHhDYfgasaacH8akY=wiFfYdH8Gipec8Eeeu0xXdbba9frFj0=OqFfea0dXdd9vqai=hGuQ8kuc9pgc9s8qqaq=dirpe0xb9q8qiLsFr0=vr0=vr0dc8meaabaqaciaacaGaaeqabaqabeGadaaakeaacuWGtbWugaqcamaaBaaaleaadaWcaaqaaiabigdaXaqaaiabd2gaTbaaaeqaaaaa@307A@, and *TLR *as a function of the excess frequency *λ *for various values of the frequency of the associated haplotype *p*_1 _= 0.1, 0.2, 0.3, 0.4 and 0.5. The remaining haplotypes were again equally frequent. We restricted ourselves to the number of observed haplotypes *m *of 5 and 8, because most of the genes can be characterized by up to 8 common variants. The parameter *λ *was varied between 0 and 0.5. The number of chromosomes *n*_1 _and *n*_2 _were 200. We used a nominal significance level of 0.05. The results are depicted in Figure 1.

For *p*_1 _= 0.5, the score statistic S^1m
 MathType@MTEF@5@5@+=feaafiart1ev1aaatCvAUfKttLearuWrP9MDH5MBPbIqV92AaeXatLxBI9gBaebbnrfifHhDYfgasaacH8akY=wiFfYdH8Gipec8Eeeu0xXdbba9frFj0=OqFfea0dXdd9vqai=hGuQ8kuc9pgc9s8qqaq=dirpe0xb9q8qiLsFr0=vr0=vr0dc8meaabaqaciaacaGaaeqabaqabeGadaaakeaacuWGtbWugaqcamaaBaaaleaadaWcaaqaaiabigdaXaqaaiabd2gaTbaaaeqaaaaa@307A@ and likelihood ratio *TLR *performed similarly, both better than Pearson's chi square. For *m *= 5 and *p*_1 _= 0.4 or 0.3 and for *m *= 8 and *p*_1 _= 0.4, 0.3 or 0.2, the score statistic S^1m
 MathType@MTEF@5@5@+=feaafiart1ev1aaatCvAUfKttLearuWrP9MDH5MBPbIqV92AaeXatLxBI9gBaebbnrfifHhDYfgasaacH8akY=wiFfYdH8Gipec8Eeeu0xXdbba9frFj0=OqFfea0dXdd9vqai=hGuQ8kuc9pgc9s8qqaq=dirpe0xb9q8qiLsFr0=vr0=vr0dc8meaabaqaciaacaGaaeqabaqabeGadaaakeaacuWGtbWugaqcamaaBaaaleaadaWcaaqaaiabigdaXaqaaiabd2gaTbaaaeqaaaaa@307A@ performed better than *TLR *especially for small *λ*. For *p*_1 _= 0.2 and *m *= 5 all three statistics had similar power. For *p*_1 _= 0.1 and *m *= 5 or *m *= 8, Pearson's chi-square performed similar to *TLR *and both statistics performed better than S^1m
 MathType@MTEF@5@5@+=feaafiart1ev1aaatCvAUfKttLearuWrP9MDH5MBPbIqV92AaeXatLxBI9gBaebbnrfifHhDYfgasaacH8akY=wiFfYdH8Gipec8Eeeu0xXdbba9frFj0=OqFfea0dXdd9vqai=hGuQ8kuc9pgc9s8qqaq=dirpe0xb9q8qiLsFr0=vr0=vr0dc8meaabaqaciaacaGaaeqabaqabeGadaaakeaacuWGtbWugaqcamaaBaaaleaadaWcaaqaaiabigdaXaqaaiabd2gaTbaaaeqaaaaa@307A@ except for *λ *≤ 0.1 and *p*_1 _= 0.1 and *m *= 5. Note that for *p*_1 _= 0.1, the pooled frequency of the associated haplotype is around 1m
 MathType@MTEF@5@5@+=feaafiart1ev1aaatCvAUfKttLearuWrP9MDH5MBPbIqV92AaeXatLxBI9gBaebbnrfifHhDYfgasaacH8akY=wiFfYdH8Gipec8Eeeu0xXdbba9frFj0=OqFfea0dXdd9vqai=hGuQ8kuc9pgc9s8qqaq=dirpe0xb9q8qiLsFr0=vr0=vr0dc8meaabaqaciaacaGaaeqabaqabeGadaaakeaadaWcaaqaaiabigdaXaqaaiabd2gaTbaaaaa@2F0F@ and hence the expectation of S^1m
 MathType@MTEF@5@5@+=feaafiart1ev1aaatCvAUfKttLearuWrP9MDH5MBPbIqV92AaeXatLxBI9gBaebbnrfifHhDYfgasaacH8akY=wiFfYdH8Gipec8Eeeu0xXdbba9frFj0=OqFfea0dXdd9vqai=hGuQ8kuc9pgc9s8qqaq=dirpe0xb9q8qiLsFr0=vr0=vr0dc8meaabaqaciaacaGaaeqabaqabeGadaaakeaacuWGtbWugaqcamaaBaaaleaadaWcaaqaaiabigdaXaqaaiabd2gaTbaaaeqaaaaa@307A@ under the alternative becomes small (see formula 4). For *m *= 5, the pooled frequency is equal to 1m
 MathType@MTEF@5@5@+=feaafiart1ev1aaatCvAUfKttLearuWrP9MDH5MBPbIqV92AaeXatLxBI9gBaebbnrfifHhDYfgasaacH8akY=wiFfYdH8Gipec8Eeeu0xXdbba9frFj0=OqFfea0dXdd9vqai=hGuQ8kuc9pgc9s8qqaq=dirpe0xb9q8qiLsFr0=vr0=vr0dc8meaabaqaciaacaGaaeqabaqabeGadaaakeaadaWcaaqaaiabigdaXaqaaiabd2gaTbaaaaa@2F0F@ = 0.2 at *λ *= 0.2 and for *m *= 8 the pooled frequency is equal to 1m
 MathType@MTEF@5@5@+=feaafiart1ev1aaatCvAUfKttLearuWrP9MDH5MBPbIqV92AaeXatLxBI9gBaebbnrfifHhDYfgasaacH8akY=wiFfYdH8Gipec8Eeeu0xXdbba9frFj0=OqFfea0dXdd9vqai=hGuQ8kuc9pgc9s8qqaq=dirpe0xb9q8qiLsFr0=vr0=vr0dc8meaabaqaciaacaGaaeqabaqabeGadaaakeaadaWcaaqaaiabigdaXaqaaiabd2gaTbaaaaa@2F0F@ = 0.125 at *λ *= 0.06. Thus when the associated haplotype has frequency around 1m
 MathType@MTEF@5@5@+=feaafiart1ev1aaatCvAUfKttLearuWrP9MDH5MBPbIqV92AaeXatLxBI9gBaebbnrfifHhDYfgasaacH8akY=wiFfYdH8Gipec8Eeeu0xXdbba9frFj0=OqFfea0dXdd9vqai=hGuQ8kuc9pgc9s8qqaq=dirpe0xb9q8qiLsFr0=vr0=vr0dc8meaabaqaciaacaGaaeqabaqabeGadaaakeaadaWcaaqaaiabigdaXaqaaiabd2gaTbaaaaa@2F0F@, the score statistic loses power.

Especially for common variants with frequency *p*_1 _of 0.3 or 0.2 and a small impact on the disease (*λ *≤ 0.1), the score statistic performed better than *TLR *statistic. For *m *= 5 and *m *= 8 and *p*_1 _= 0.3, the gain in power of the score statistic compared to *TLR *statistic was about 4% and 8% for *λ *of 0.05 and 0.1 respectively. For *m *= 5 and *p*_1 _= 0.2, both statistics performed similar. For *m *= 8 and *p*_1 _= 0.2 the gain in power of the score statistic was large, namely 7% and 12% for *λ *of 0.05 and 0.1 respectively.

### Data example

We applied the three statistics to a study on haplotype association of fibrinogen alpha (FGA), beta (FGB) and gamma (FGG) with risk of deep venous thrombosis in LETS [15,16]. Fifteen haplotype tagging SNPs were typed in 474 cases and 474 controls [9]. Within the three genes, the SNPs were in high linkage disequilibrium (rh2
 MathType@MTEF@5@5@+=feaafiart1ev1aaatCvAUfKttLearuWrP9MDH5MBPbIqV92AaeXatLxBI9gBaebbnrfifHhDYfgasaacH8akY=wiFfYdH8Gipec8Eeeu0xXdbba9frFj0=OqFfea0dXdd9vqai=hGuQ8kuc9pgc9s8qqaq=dirpe0xb9q8qiLsFr0=vr0=vr0dc8meaabaqaciaacaGaaeqabaqabeGadaaakeaacqWGYbGCdaqhaaWcbaGaemiAaGgabaGaeGOmaidaaaaa@3091@ > 0.95). The number of common haplotypes (frequency greater than 5%) describing FGG, FGA and FGB were three, five and five respectively. Since we focus on common haplotypes, we pooled the rare haplotypes with similar haplotypes by dropping rare tagging SNPs. For FGG, we pooled H4 with H1 and for FGB, we pooled H5 with H4 and H7 with H3 (see Uitte de Willige et al. [9] for more details on the tagging SNPs). In this analysis we considered p-values below 0.05 to be significant. In Table 2 the data are described and the results are given.

**Table 2:** Descriptives and results of genetic association on LETS

haplotype	case chromosomes (%)	control chromosomes (%)	*X*^2^	S^1m MathType@MTEF@5@5@+=feaafiart1ev1aaatCvAUfKttLearuWrP9MDH5MBPbIqV92AaeXatLxBI9gBaebbnrfifHhDYfgasaacH8akY=wiFfYdH8Gipec8Eeeu0xXdbba9frFj0=OqFfea0dXdd9vqai=hGuQ8kuc9pgc9s8qqaq=dirpe0xb9q8qiLsFr0=vr0=vr0dc8meaabaqaciaacaGaaeqabaqabeGadaaakeaacuWGtbWugaqcamaaBaaaleaadaWcaaqaaiabigdaXaqaaiabd2gaTbaaaeqaaaaa@307A@	TLR	λ^ MathType@MTEF@5@5@+=feaafiart1ev1aaatCvAUfKttLearuWrP9MDH5MBPbIqV92AaeXatLxBI9gBaebbnrfifHhDYfgasaacH8akY=wiFfYdH8Gipec8Eeeu0xXdbba9frFj0=OqFfea0dXdd9vqai=hGuQ8kuc9pgc9s8qqaq=dirpe0xb9q8qiLsFr0=vr0=vr0dc8meaabaqaciaacaGaaeqabaqabeGadaaakeaaiiGacuWF7oaBgaqcaaaa@2E77@
FGG (*n*_1 _= 938, *n*_2 _= 942)			0.005	0.515	0.003	0.09

H1 + H4	374 (39.8)	399 (42.4)				
H2	311 (33.7)	254 (26.9)				
H3	249 (26.5)	289 (30.7)				

FGA (*n*_1 _= 936, *n*_2 _= 942)			0.034	0.003	0.012	0.07

H1	270 (28.7)	266 (28.2)				
H2	326 (34.5)	270 (28.7)				
H3	95 (10.1)	117 (12.4)				
H4	100 (10.6)	121 (12.8)				
H5	151 (16.1)	168 (17.8)				

FGB (*n*_1 _= 936, *n*_2 _= 932)			0.090	0.049	0.072	0.05

H1	329 (34.9)	316 (33.6)				
H2	232 (24.6)	190 (20.2)				
H4+H5	148 (15.7)	166 (17.7)				
H6	128 (13.6)	143 (15.2)				
H3+H7	105 (11.1)	125 (13.3)				

For all genes, haplotype H2 appeared to be more frequent in the cases than in the controls. For FGG, FGA and FGB the allelic odds ratios of presence of H2 versus the rest was 1.34, 1.29 and 1.28 respectively. Note that these odds ratios were rather similar while the p-values of the corresponding chi square statistics were different namely, 0.005, 0.034 and 0.090 respectively. The difference in p-values was caused by the difference in degrees of freedom of the chi square statistics and the frequencies of other haplotypes. From the results of the standard chi-square statistics we concluded that only FGG was significantly associated to thrombophilia.

The p-values of the *TLR *were respectively 0.003, 0.012 and 0.072. These p-values were in line with the estimates of *λ*, namely 0.08, 0.07 and 0.05 respectively. Since FGA and FGB both had 5 variants, the frequency of the associated haplotype H2 was 0.3 and 0.2 respectively and the *λ*'s were rather small, the score statistic should have more power than *TLR *for these genes. On the other hand *TLR *may perform better on the FGG gene, because of the larger difference in frequency between cases and controls and because the frequencies in the pooled set are around 1/*m*. For this situation, the score statistic S^1m
 MathType@MTEF@5@5@+=feaafiart1ev1aaatCvAUfKttLearuWrP9MDH5MBPbIqV92AaeXatLxBI9gBaebbnrfifHhDYfgasaacH8akY=wiFfYdH8Gipec8Eeeu0xXdbba9frFj0=OqFfea0dXdd9vqai=hGuQ8kuc9pgc9s8qqaq=dirpe0xb9q8qiLsFr0=vr0=vr0dc8meaabaqaciaacaGaaeqabaqabeGadaaakeaacuWGtbWugaqcamaaBaaaleaadaWcaaqaaiabigdaXaqaaiabd2gaTbaaaeqaaaaa@307A@ has little power.

The p-values of the score statistic S^1m
 MathType@MTEF@5@5@+=feaafiart1ev1aaatCvAUfKttLearuWrP9MDH5MBPbIqV92AaeXatLxBI9gBaebbnrfifHhDYfgasaacH8akY=wiFfYdH8Gipec8Eeeu0xXdbba9frFj0=OqFfea0dXdd9vqai=hGuQ8kuc9pgc9s8qqaq=dirpe0xb9q8qiLsFr0=vr0=vr0dc8meaabaqaciaacaGaaeqabaqabeGadaaakeaacuWGtbWugaqcamaaBaaaleaadaWcaaqaaiabigdaXaqaaiabd2gaTbaaaeqaaaaa@307A@ were 0.515, 0.003 and 0.049 for FGG, FGA and FGB respectively. Indeed the p-values for FGA and FGB were smaller than the corresponding p-values of *TLR *statistic and the score statistic had no power for FGA. Based on the results of the score statistic, FGA and FGB were significantly associated with thrombophilia risk.

Although for each subject we were able to derive the pair of haplotypes, we also estimated the p-values of the statistics by 1000 permutations using TAGSNPS to estimate the haplotype counts in each permutation step. The results were similar.

## Discussion

In this report we have derived a new score statistic to test for association between a candidate gene and a binary trait. Score statistics are easy to compute, locally most powerful, and robust to small model deviations under the alternative [7]. From our simulations it appears that under the null hypothesis the statistic follows a chi-square distribution. For candidate genes with a small impact on the disease (*λ *small), five to eight observed variants, and a frequency of the associated variant in the range of 0.2 to 0.4, the new statistic performs better than the Terwilliger's LR statistic. For larger frequencies (around 0.5) or for a large number of variants (*m *> 8), Terwilliger's LR statistic appears to perform better, because the weight corresponding to the associated haplotype is larger. Compared to Pearson's chi-square statistic, the score statistic has more power when the associated haplotype has frequency larger than 0.2 and the number of observed variants *m *is larger than 5. Especially when the frequency of the associated haplotype is small (< 0.2), Pearson chi-squared statistic performs similar or better than the score statistic.

We used a mixture of two chi-square distributions to approximate the distribution of Terwilliger's LR statistic under the null hypothesis. It is known that this distribution appears to give conservative p-values [6]. Permutations of the case-control status may result in better p-values. However, permutations are not attractive when no simple formulae for the statistic exists.

When the sampled population is not in Hardy Weinberg equilibrium, the considered statistics are not valid since they assume that the haplotypes of a subject are independent. For Pearson's chi-square statistic on haplotypes, a multi-allelic version of Armitage's trend test on pairs of haplotypes is available [17,18]. Under Hardy Weinberg equilibrium these statistics are asymptotically equivalent. To derive a one degree of freedom score statistic for pairs of haplotypes, a logistic regression model can be used with the number of associated haplotypes as covariate. Then the total likelihood is the average of the likelihoods over these logistic regression models of possible associated haplotypes. Here more research is needed. In this paper we used a smoothing approach to deal with the fact that the associated haplotype is unknown. As alternative, one may consider taking the maximum over each haplotype. A disadvantage of the latter approach is that permutations are needed to derive a p-value [19].

In this paper we assumed that the haplotypes could be derived from the typed tagging SNPs. When uncertainty in phase exists the haplotype frequencies have to be estimated using for example an EM algorithm [20] and the uncertainty has to be taken into account when the p-values are derived. This can easily be done by using permutations. Our program [11] is able to compute valid p-values in case of ambiguous phase by calling TAGSNPS [8] to derive the haplotype counts in each permutation step. It may be more efficient to incorporate phase unambiguity in the likelihood function. The gain in efficiency will probably be small. Analyzing genes with little information on phase yields many relatively rare haplotypes and interpretation of the results is often hard. Using permutations to deal with haplotype uncertainty is in the line with recent developments in weighted regression analysis [21] and in imputation of haplotypes [22]. The new score statistic has no power when the frequency of the associated haplotype in the pooled set of cases and controls is around 1m
 MathType@MTEF@5@5@+=feaafiart1ev1aaatCvAUfKttLearuWrP9MDH5MBPbIqV92AaeXatLxBI9gBaebbnrfifHhDYfgasaacH8akY=wiFfYdH8Gipec8Eeeu0xXdbba9frFj0=OqFfea0dXdd9vqai=hGuQ8kuc9pgc9s8qqaq=dirpe0xb9q8qiLsFr0=vr0=vr0dc8meaabaqaciaacaGaaeqabaqabeGadaaakeaadaWcaaqaaiabigdaXaqaaiabd2gaTbaaaaa@2F0F@. This raises the question if a better summary statistic of Pearson's chi square statistic into an one degree of freedom statistic exists. Here more research is needed. As an alternative for the score function, the second derivative of the log likelihood may be used [23]. In another paper we study the performance of this second derivative of the log likelihood function of Terwilliger [24].

## Conclusion

We conclude that by choosing alternative weights, in particular constant weights, in the likelihood of Terwilliger, a set of new powerful and robust statistical tests was derived. For genetic association studies aiming to identify common associated variants, the new statistic should be used in addition to the standard Pearson's chi square statistic. By using both statistics more insight in the data can be obtained. A program [11] which computes the statistics and corresponding p-values is freely available.

## Methods

Let *m *be the number of haplotypes describing most of the genetic variation in a gene. Assume that the haplotype frequencies are in Hardy-Weinberg equilibrium proportions. Let *p *= (*p*_1_,⋯, *p*_*m*_) be the vector of haplotype frequencies in controls. Assume that only one haplotype denoted with index *i *is over-represented in the cases, then the haplotype frequencies in the cases can be modelled as *q*_*i *_= *p*_*i *_+ *λ*(1 - *p*_*i*_) and *q*_*j *_= *p*_*j *_- *λp*_*j *_for *j *∈ (1,⋯,*i *- 1, *i *+ 1,⋯,*m*). Let *x *= (*x*_1_,⋯,*x*_*m*_) and *y *= (*y*_1_,⋯,*y*_*m*_) be vectors of haplotype counts in the cases and the controls, respectively, and let *n*_1 _and *n*_2 _be the total number of case chromosomes and of control chromosomes, respectively, and *n *= *n*_1 _+ *n*_2_. Then the conditional likelihood *L*_*i *_given that haplotype *i *carries the mutation is given by

Li(λ,p|x,y)=(pi+λ(1−pi))xi(1−λ)n1−xi∏j≠impjxj∏j=1mpjyj
 MathType@MTEF@5@5@+=feaafiart1ev1aaatCvAUfKttLearuWrP9MDH5MBPbIqV92AaeXatLxBI9gBaebbnrfifHhDYfgasaacH8akY=wiFfYdH8Gipec8Eeeu0xXdbba9frFj0=OqFfea0dXdd9vqai=hGuQ8kuc9pgc9s8qqaq=dirpe0xb9q8qiLsFr0=vr0=vr0dc8meaabaqaciaacaGaaeqabaqabeGadaaakeaacqWGmbatdaWgaaWcbaGaemyAaKgabeaakiabcIcaOGGaciab=T7aSjabcYcaSiabdchaWjabcYha8jabdIha4jabcYcaSiabdMha5jabcMcaPiabg2da9iabcIcaOiabdchaWnaaBaaaleaacqWGPbqAaeqaaOGaey4kaSIae83UdWMaeiikaGIaeGymaeJaeyOeI0IaemiCaa3aaSbaaSqaaiabdMgaPbqabaGccqGGPaqkcqGGPaqkdaahaaWcbeqaaiabdIha4naaBaaameaacqWGPbqAaeqaaaaakiabcIcaOiabigdaXiabgkHiTiab=T7aSjabcMcaPmaaCaaaleqabaGaemOBa42aaSbaaWqaaiabigdaXaqabaWccqGHsislcqWG4baEdaWgaaadbaGaemyAaKgabeaaaaGcdaqeWbqaaiabdchaWnaaDaaaleaacqWGQbGAaeaacqWG4baEdaWgaaadbaGaemOAaOgabeaaaaaaleaacqWGQbGAcqGHGjsUcqWGPbqAaeaacqWGTbqBa0Gaey4dIunakmaarahabaGaemiCaa3aa0baaSqaaiabdQgaQbqaaiabdMha5naaBaaameaacqWGQbGAaeqaaaaaaSqaaiabdQgaQjabg2da9iabigdaXaqaaiabd2gaTbqdcqGHpis1aaaa@734E@

and the likelihood proposed by Terwilliger is equal to

L(λ,p|x,y)=∑j=1mpjLj,
 MathType@MTEF@5@5@+=feaafiart1ev1aaatCvAUfKttLearuWrP9MDH5MBPbIqV92AaeXatLxBI9gBaebbnrfifHhDYfgasaacH8akY=wiFfYdH8Gipec8Eeeu0xXdbba9frFj0=OqFfea0dXdd9vqai=hGuQ8kuc9pgc9s8qqaq=dirpe0xb9q8qiLsFr0=vr0=vr0dc8meaabaqaciaacaGaaeqabaqabeGadaaakeaacqWGmbatcqGGOaakiiGacqWF7oaBcqGGSaalcqWGWbaCcqGG8baFcqWG4baEcqGGSaalcqWG5bqEcqGGPaqkcqGH9aqpdaaeWbqaaiabdchaWnaaBaaaleaacqWGQbGAaeqaaOGaemitaW0aaSbaaSqaaiabdQgaQbqabaaabaGaemOAaOMaeyypa0JaeGymaedabaGaemyBa0ganiabggHiLdGccqGGSaalaaa@475A@

with *L*_*j *_given in formula (1).

It is easy to see that likelihood function (2) can be generalized to the following likelihood function:

L(λ,p|x,y,w)=∑j=1mwjLj,
 MathType@MTEF@5@5@+=feaafiart1ev1aaatCvAUfKttLearuWrP9MDH5MBPbIqV92AaeXatLxBI9gBaebbnrfifHhDYfgasaacH8akY=wiFfYdH8Gipec8Eeeu0xXdbba9frFj0=OqFfea0dXdd9vqai=hGuQ8kuc9pgc9s8qqaq=dirpe0xb9q8qiLsFr0=vr0=vr0dc8meaabaqaciaacaGaaeqabaqabeGadaaakeaacqWGmbatcqGGOaakiiGacqWF7oaBcqGGSaalcqWGWbaCcqGG8baFcqWG4baEcqGGSaalcqWG5bqEcqGGSaalcqWG3bWDcqGGPaqkcqGH9aqpdaaeWbqaaiabdEha3naaBaaaleaacqWGQbGAaeqaaOGaemitaW0aaSbaaSqaaiabdQgaQbqabaaabaGaemOAaOMaeyypa0JaeGymaedabaGaemyBa0ganiabggHiLdGccqGGSaalaaa@49BF@

with *L*_*j *_given in formula (1) and *w *= (*w*_1_,⋯,*w*_*m*_) a vector of known positive weights restricted by ∑j=1mwj=1
 MathType@MTEF@5@5@+=feaafiart1ev1aaatCvAUfKttLearuWrP9MDH5MBPbIqV92AaeXatLxBI9gBaebbnrfifHhDYfgasaacH8akY=wiFfYdH8Gipec8Eeeu0xXdbba9frFj0=OqFfea0dXdd9vqai=hGuQ8kuc9pgc9s8qqaq=dirpe0xb9q8qiLsFr0=vr0=vr0dc8meaabaqaciaacaGaaeqabaqabeGadaaakeaadaaeWaqaaiabdEha3naaBaaaleaacqWGQbGAaeqaaOGaeyypa0JaeGymaedaleaacqWGQbGAcqGH9aqpcqaIXaqmaeaacqWGTbqBa0GaeyyeIuoaaaa@3864@. The first derivative of the log likelihood *l*(*λ*, *p*|*x*, *y*, *w*) = log(*L*(*λ*, *p*|*x*, *y*, *w*)) to *λ *evaluated in *λ *= 0 is equal to

Uw=∂∂λl(λ,p|x,y,w)|λ=0=∑j=1mwj(xj−n1pj)pj.
 MathType@MTEF@5@5@+=feaafiart1ev1aaatCvAUfKttLearuWrP9MDH5MBPbIqV92AaeXatLxBI9gBaebbnrfifHhDYfgasaacH8akY=wiFfYdH8Gipec8Eeeu0xXdbba9frFj0=OqFfea0dXdd9vqai=hGuQ8kuc9pgc9s8qqaq=dirpe0xb9q8qiLsFr0=vr0=vr0dc8meaabaqaciaacaGaaeqabaqabeGadaaakeaafaqadeGabaaabaGaemyvau1aaSbaaSqaaiabdEha3bqabaGccqGH9aqpdaWcaaqaaiabgkGi2cqaaiabgkGi2IGaciab=T7aSbaacqWGSbaBcqGGOaakcqWF7oaBcqGGSaalcqWGWbaCcqGG8baFcqWG4baEcqGGSaalcqWG5bqEcqGGSaalcqWG3bWDcqGGPaqkdaWgaaWcbaGaeiiFaWNae83UdWMaeyypa0JaeGimaadabeaaaOqaaiabg2da9maaqahabaWaaSaaaeaacqWG3bWDdaWgaaWcbaGaemOAaOgabeaakiabcIcaOiabdIha4naaBaaaleaacqWGQbGAaeqaaOGaeyOeI0IaemOBa42aaSbaaSqaaiabigdaXaqabaGccqWGWbaCdaWgaaWcbaGaemOAaOgabeaakiabcMcaPaqaaiabdchaWnaaBaaaleaacqWGQbGAaeqaaaaakiabc6caUaWcbaGaemOAaOMaeyypa0JaeGymaedabaGaemyBa0ganiabggHiLdaaaaaa@6371@

For known allele frequencies *p*_*j*_, the distribution of the *U*_*w *_under *H*_0 _can be approximated by the normal distribution with zero mean and variance VAR[Uw]=n1(∑j=1mwj2pj−1−1)
 MathType@MTEF@5@5@+=feaafiart1ev1aaatCvAUfKttLearuWrP9MDH5MBPbIqV92AaeXatLxBI9gBaebbnrfifHhDYfgasaacH8akY=wiFfYdH8Gipec8Eeeu0xXdbba9frFj0=OqFfea0dXdd9vqai=hGuQ8kuc9pgc9s8qqaq=dirpe0xb9q8qiLsFr0=vr0=vr0dc8meaabaqaciaacaGaaeqabaqabeGadaaakeaacqqGwbGvcqqGbbqqcqqGsbGucqGGBbWwcqWGvbqvdaWgaaWcbaGaem4DaChabeaakiabc2faDjabg2da9iabd6gaUnaaBaaaleaacqaIXaqmaeqaaOGaeiikaGYaaabmaeaacqWG3bWDdaqhaaWcbaGaemOAaOgabaGaeGOmaidaaOGaemiCaa3aa0baaSqaaiabdQgaQbqaaiabgkHiTiabikdaYaaakiabgkHiTiabigdaXiabcMcaPaWcbaGaemOAaOMaeyypa0JaeGymaedabaGaemyBa0ganiabggHiLdaaaa@4C24@. Note that *U*_*w *_= 0 when for all *j w*_*j *_= *p*_*j*_.

Often the haplotype frequencies are unknown and have to be estimated from the data. Under the null hypothesis we can estimate the frequencies from the combined sample of cases and controls by p^j=xj+yjn
 MathType@MTEF@5@5@+=feaafiart1ev1aaatCvAUfKttLearuWrP9MDH5MBPbIqV92AaeXatLxBI9gBaebbnrfifHhDYfgasaacH8akY=wiFfYdH8Gipec8Eeeu0xXdbba9frFj0=OqFfea0dXdd9vqai=hGuQ8kuc9pgc9s8qqaq=dirpe0xb9q8qiLsFr0=vr0=vr0dc8meaabaqaciaacaGaaeqabaqabeGadaaakeaacuWGWbaCgaqcamaaBaaaleaacqWGQbGAaeqaaOGaeyypa0ZaaSaaaeaacqWG4baEdaWgaaWcbaGaemOAaOgabeaakiabgUcaRiabdMha5naaBaaaleaacqWGQbGAaeqaaaGcbaGaemOBa4gaaaaa@392F@ and an estimate of the score statistic *U*_*w *_is given by

U^w=∑j=1mwj(xj−n1p^j)p^j.
 MathType@MTEF@5@5@+=feaafiart1ev1aaatCvAUfKttLearuWrP9MDH5MBPbIqV92AaeXatLxBI9gBaebbnrfifHhDYfgasaacH8akY=wiFfYdH8Gipec8Eeeu0xXdbba9frFj0=OqFfea0dXdd9vqai=hGuQ8kuc9pgc9s8qqaq=dirpe0xb9q8qiLsFr0=vr0=vr0dc8meaabaqaciaacaGaaeqabaqabeGadaaakeaacuWGvbqvgaqcamaaBaaaleaacqWG3bWDaeqaaOGaeyypa0ZaaabCaeaadaWcaaqaaiabdEha3naaBaaaleaacqWGQbGAaeqaaOGaeiikaGIaemiEaG3aaSbaaSqaaiabdQgaQbqabaGccqGHsislcqWGUbGBdaWgaaWcbaGaeGymaedabeaakiqbdchaWzaajaWaaSbaaSqaaiabdQgaQbqabaGccqGGPaqkaeaacuWGWbaCgaqcamaaBaaaleaacqWGQbGAaeqaaaaaaeaacqWGQbGAcqGH9aqpcqaIXaqmaeaacqWGTbqBa0GaeyyeIuoakiabc6caUaaa@49DB@

under *H*_0_, U^w
 MathType@MTEF@5@5@+=feaafiart1ev1aaatCvAUfKttLearuWrP9MDH5MBPbIqV92AaeXatLxBI9gBaebbnrfifHhDYfgasaacH8akY=wiFfYdH8Gipec8Eeeu0xXdbba9frFj0=OqFfea0dXdd9vqai=hGuQ8kuc9pgc9s8qqaq=dirpe0xb9q8qiLsFr0=vr0=vr0dc8meaabaqaciaacaGaaeqabaqabeGadaaakeaacuWGvbqvgaqcamaaBaaaleaacqWG3bWDaeqaaaaa@2F92@ has approximately mean equal to zero and variance

VAR(U^w)≈n−1n1n2(∑j=1mwj2pj−1−1).
 MathType@MTEF@5@5@+=feaafiart1ev1aaatCvAUfKttLearuWrP9MDH5MBPbIqV92AaeXatLxBI9gBaebbnrfifHhDYfgasaacH8akY=wiFfYdH8Gipec8Eeeu0xXdbba9frFj0=OqFfea0dXdd9vqai=hGuQ8kuc9pgc9s8qqaq=dirpe0xb9q8qiLsFr0=vr0=vr0dc8meaabaqaciaacaGaaeqabaqabeGadaaakeaacqqGwbGvcqqGbbqqcqqGsbGucqGGOaakcuWGvbqvgaqcamaaBaaaleaacqWG3bWDaeqaaOGaeiykaKIaeyisISRaemOBa42aaWbaaSqabeaacqGHsislcqaIXaqmaaGccqWGUbGBdaWgaaWcbaGaeGymaedabeaakiabd6gaUnaaBaaaleaacqaIYaGmaeqaaOGaeiikaGYaaabCaeaacqWG3bWDdaqhaaWcbaGaemOAaOgabaGaeGOmaidaaOGaemiCaa3aa0baaSqaaiabdQgaQbqaaiabgkHiTiabigdaXaaakiabgkHiTiabigdaXiabcMcaPiabc6caUaWcbaGaemOAaOMaeyypa0JaeGymaedabaGaemyBa0ganiabggHiLdaaaa@5339@

Note that the variance VAR(U^w)
 MathType@MTEF@5@5@+=feaafiart1ev1aaatCvAUfKttLearuWrP9MDH5MBPbIqV92AaeXatLxBI9gBaebbnrfifHhDYfgasaacH8akY=wiFfYdH8Gipec8Eeeu0xXdbba9frFj0=OqFfea0dXdd9vqai=hGuQ8kuc9pgc9s8qqaq=dirpe0xb9q8qiLsFr0=vr0=vr0dc8meaabaqaciaacaGaaeqabaqabeGadaaakeaacqqGwbGvcqqGbbqqcqqGsbGucqGGOaakcuWGvbqvgaqcamaaBaaaleaacqWG3bWDaeqaaOGaeiykaKcaaa@34B5@ is increased by *n*_2_/*n *fold compared to the variance VAR(*U*_*w*_) because the allele frequencies are estimated from the data. Now the score statistic S^w
 MathType@MTEF@5@5@+=feaafiart1ev1aaatCvAUfKttLearuWrP9MDH5MBPbIqV92AaeXatLxBI9gBaebbnrfifHhDYfgasaacH8akY=wiFfYdH8Gipec8Eeeu0xXdbba9frFj0=OqFfea0dXdd9vqai=hGuQ8kuc9pgc9s8qqaq=dirpe0xb9q8qiLsFr0=vr0=vr0dc8meaabaqaciaacaGaaeqabaqabeGadaaakeaacuWGtbWugaqcamaaBaaaleaacqWG3bWDaeqaaaaa@2F8E@ is defined by

S^w=U^w2VA^R(U^w),
 MathType@MTEF@5@5@+=feaafiart1ev1aaatCvAUfKttLearuWrP9MDH5MBPbIqV92AaeXatLxBI9gBaebbnrfifHhDYfgasaacH8akY=wiFfYdH8Gipec8Eeeu0xXdbba9frFj0=OqFfea0dXdd9vqai=hGuQ8kuc9pgc9s8qqaq=dirpe0xb9q8qiLsFr0=vr0=vr0dc8meaabaqaciaacaGaaeqabaqabeGadaaakeaacuWGtbWugaqcamaaBaaaleaacqWG3bWDaeqaaOGaeyypa0ZaaSaaaeaacuWGvbqvgaqcamaaDaaaleaacqWG3bWDaeaacqaIYaGmaaaakeaacqqGwbGvcuqGbbqqgaqcaiabbkfasjabcIcaOiqbdwfavzaajaWaaSbaaSqaaiabdEha3bqabaGccqGGPaqkaaGaeiilaWcaaa@3D8A@

where VA^R(U^w)
 MathType@MTEF@5@5@+=feaafiart1ev1aaatCvAUfKttLearuWrP9MDH5MBPbIqV92AaeXatLxBI9gBaebbnrfifHhDYfgasaacH8akY=wiFfYdH8Gipec8Eeeu0xXdbba9frFj0=OqFfea0dXdd9vqai=hGuQ8kuc9pgc9s8qqaq=dirpe0xb9q8qiLsFr0=vr0=vr0dc8meaabaqaciaacaGaaeqabaqabeGadaaakeaacqqGwbGvcuqGbbqqgaqcaiabbkfasjabcIcaOiqbdwfavzaajaWaaSbaaSqaaiabdEha3bqabaGccqGGPaqkaaa@34C5@ is obtained by replacing *p*_*j *_by its estimate p^j
 MathType@MTEF@5@5@+=feaafiart1ev1aaatCvAUfKttLearuWrP9MDH5MBPbIqV92AaeXatLxBI9gBaebbnrfifHhDYfgasaacH8akY=wiFfYdH8Gipec8Eeeu0xXdbba9frFj0=OqFfea0dXdd9vqai=hGuQ8kuc9pgc9s8qqaq=dirpe0xb9q8qiLsFr0=vr0=vr0dc8meaabaqaciaacaGaaeqabaqabeGadaaakeaacuWGWbaCgaqcamaaBaaaleaacqWGQbGAaeqaaaaa@2FAE@ in formula (3). When all haplotypes are common, a natural choice of weights is wj=1m
 MathType@MTEF@5@5@+=feaafiart1ev1aaatCvAUfKttLearuWrP9MDH5MBPbIqV92AaeXatLxBI9gBaebbnrfifHhDYfgasaacH8akY=wiFfYdH8Gipec8Eeeu0xXdbba9frFj0=OqFfea0dXdd9vqai=hGuQ8kuc9pgc9s8qqaq=dirpe0xb9q8qiLsFr0=vr0=vr0dc8meaabaqaciaacaGaaeqabaqabeGadaaakeaacqWG3bWDdaWgaaWcbaGaemOAaOgabeaakiabg2da9maalaaabaGaeGymaedabaGaemyBa0gaaaaa@331F@.

Under the alternative hypothesis of the presence of one positively associated haplotype *i*, the expectation of U^1m
 MathType@MTEF@5@5@+=feaafiart1ev1aaatCvAUfKttLearuWrP9MDH5MBPbIqV92AaeXatLxBI9gBaebbnrfifHhDYfgasaacH8akY=wiFfYdH8Gipec8Eeeu0xXdbba9frFj0=OqFfea0dXdd9vqai=hGuQ8kuc9pgc9s8qqaq=dirpe0xb9q8qiLsFr0=vr0=vr0dc8meaabaqaciaacaGaaeqabaqabeGadaaakeaacuWGvbqvgaqcamaaBaaaleaadaWcaaqaaiabigdaXaqaaiabd2gaTbaaaeqaaaaa@307E@ is

EHA[U^1m]≈n1n2λn−n1λ(nn1qi+n2pi−m),
 MathType@MTEF@5@5@+=feaafiart1ev1aaatCvAUfKttLearuWrP9MDH5MBPbIqV92AaeXatLxBI9gBaebbnrfifHhDYfgasaacH8akY=wiFfYdH8Gipec8Eeeu0xXdbba9frFj0=OqFfea0dXdd9vqai=hGuQ8kuc9pgc9s8qqaq=dirpe0xb9q8qiLsFr0=vr0=vr0dc8meaabaqaciaacaGaaeqabaqabeGadaaakeaacqWGfbqrdaWgaaWcbaGaemisaG0aaSbaaWqaaiabdgeabbqabaaaleqaaOWaamWaaeaacuWGvbqvgaqcamaaBaaaleaadaWcaaqaaiabigdaXaqaaiabd2gaTbaaaeqaaaGccaGLBbGaayzxaaGaeyisIS7aaSaaaeaacqWGUbGBdaWgaaWcbaGaeGymaedabeaakiabd6gaUnaaBaaaleaacqaIYaGmaeqaaGGacOGae83UdWgabaGaemOBa4MaeyOeI0IaemOBa42aaSbaaSqaaiabigdaXaqabaGccqWF7oaBaaGaeiikaGYaaSaaaeaacqWGUbGBaeaacqWGUbGBdaWgaaWcbaGaeGymaedabeaakiabdghaXnaaBaaaleaacqWGPbqAaeqaaOGaey4kaSIaemOBa42aaSbaaSqaaiabikdaYaqabaGccqWGWbaCdaWgaaWcbaGaemyAaKgabeaaaaGccqGHsislcqWGTbqBcqGGPaqkcqGGSaalaaa@5786@

with *q*_*i *_= *p*_*i *_+ *λ*(1 - *p*_*i*_). Note that n1pi+n2qin
 MathType@MTEF@5@5@+=feaafiart1ev1aaatCvAUfKttLearuWrP9MDH5MBPbIqV92AaeXatLxBI9gBaebbnrfifHhDYfgasaacH8akY=wiFfYdH8Gipec8Eeeu0xXdbba9frFj0=OqFfea0dXdd9vqai=hGuQ8kuc9pgc9s8qqaq=dirpe0xb9q8qiLsFr0=vr0=vr0dc8meaabaqaciaacaGaaeqabaqabeGadaaakeaadaWcaaqaaiabd6gaUnaaBaaaleaacqaIXaqmaeqaaOGaemiCaa3aaSbaaSqaaiabdMgaPbqabaGccqGHRaWkcqWGUbGBdaWgaaWcbaGaeGOmaidabeaakiabdghaXnaaBaaaleaacqWGPbqAaeqaaaGcbaGaemOBa4gaaaaa@3A11@ is the frequency of the associated haplotype in the combined sample. When n1pi+n2qin=1m
 MathType@MTEF@5@5@+=feaafiart1ev1aaatCvAUfKttLearuWrP9MDH5MBPbIqV92AaeXatLxBI9gBaebbnrfifHhDYfgasaacH8akY=wiFfYdH8Gipec8Eeeu0xXdbba9frFj0=OqFfea0dXdd9vqai=hGuQ8kuc9pgc9s8qqaq=dirpe0xb9q8qiLsFr0=vr0=vr0dc8meaabaqaciaacaGaaeqabaqabeGadaaakeaadaWcaaqaaiabd6gaUnaaBaaaleaacqaIXaqmaeqaaOGaemiCaa3aaSbaaSqaaiabdMgaPbqabaGccqGHRaWkcqWGUbGBdaWgaaWcbaGaeGOmaidabeaakiabdghaXnaaBaaaleaacqWGPbqAaeqaaaGcbaGaemOBa4gaaiabg2da9maalaaabaGaeGymaedabaGaemyBa0gaaaaa@3D7A@, the expectation of the score statistic is equal to 0. Hence the score statistic has little power. When n1pi+n2qin
 MathType@MTEF@5@5@+=feaafiart1ev1aaatCvAUfKttLearuWrP9MDH5MBPbIqV92AaeXatLxBI9gBaebbnrfifHhDYfgasaacH8akY=wiFfYdH8Gipec8Eeeu0xXdbba9frFj0=OqFfea0dXdd9vqai=hGuQ8kuc9pgc9s8qqaq=dirpe0xb9q8qiLsFr0=vr0=vr0dc8meaabaqaciaacaGaaeqabaqabeGadaaakeaadaWcaaqaaiabd6gaUnaaBaaaleaacqaIXaqmaeqaaOGaemiCaa3aaSbaaSqaaiabdMgaPbqabaGccqGHRaWkcqWGUbGBdaWgaaWcbaGaeGOmaidabeaakiabdghaXnaaBaaaleaacqWGPbqAaeqaaaGcbaGaemOBa4gaaaaa@3A11@ is larger than 1m
 MathType@MTEF@5@5@+=feaafiart1ev1aaatCvAUfKttLearuWrP9MDH5MBPbIqV92AaeXatLxBI9gBaebbnrfifHhDYfgasaacH8akY=wiFfYdH8Gipec8Eeeu0xXdbba9frFj0=OqFfea0dXdd9vqai=hGuQ8kuc9pgc9s8qqaq=dirpe0xb9q8qiLsFr0=vr0=vr0dc8meaabaqaciaacaGaaeqabaqabeGadaaakeaadaWcaaqaaiabigdaXaqaaiabd2gaTbaaaaa@2F0F@ the expectation becomes negative. Therefore we propose a chi-square distribution with one degree of freedom to approximate the distribution of this statistic under the null hypothesis.

## Abbreviations

LR: Likelihood ratio with equal weights

TLR: Terwilliger's likelihood ratio

FGA: fibrinogen alpha

FGB: fibrinogen beta

FGG: fibrinogen gamma

SNP: single nucleotide polymorphism

LETS: the Leiden thrombophilia study

## Authors' contributions

REG participated in method development, wrote a C code, carried out the simulation study and data analysis, participated in interpreting results, and in drafting of the manuscript. SUdeW and MCHdeV provided the data and participated in interpreting the results. QH designed the web interface and extended the C code to account for phase uncertainty. LH participated in method development and in interpreting results. JJH-D participated in method development and in interpreting results, and participate in drafting of the manuscript. All authors read and approved the final version.
